# Two less common human microRNAs miR-875 and miR-3144 target a conserved site of E6 oncogene in most high-risk human papillomavirus subtypes

**DOI:** 10.1007/s13238-015-0142-8

**Published:** 2015-04-28

**Authors:** Lin Lin, Qingqing Cai, Xiaoyan Zhang, Hongwei Zhang, Yang Zhong, Congjian Xu, Yanyun Li

**Affiliations:** 1 Department of Gynecology, Obstetrics and Gynecology Hospital, Fudan University, Shanghai, 200011 China; 2 Department of Obstetrics and Gynecology of Shanghai Medical School, Fudan University, Shanghai, 200032 China; 3 Shanghai Key Laboratory of Female Reproductive Endocrine Related Diseases, Shanghai, 200011 China; 4 Institute of Biomedical Sciences, Fudan University, Shanghai, 200032 China; 5 School of Life Sciences and Technology, Tongji University, Shanghai, 200092 China; 6 Shanghai Center for Bioinformation Technology, Shanghai, 200235 China; 7 School of Life Sciences, Fudan University, Shanghai, 200433 China

**Keywords:** human papillomavirus, microRNA, E6, miR-875, miR-3144

## Abstract

Human papillomaviruses (HPVs) including high-risk (HR) and low-risk (LR) subtypes have distinguishable variation on both genotypes and phenotypes. The co-infection of multiple HR-HPVs, headed by HPV16, is common in cervical cancer in female. Recently accumulating reports have focused on the interaction between virus and host, particularly the role of human microRNAs (miRNAs) in anti-viral defense by targeting viral genome. Here, we found a well-conserved target site of miRNAs in the genomes of most HR-HPVs, not LR-HPVs, by scanning all potential target sites of human miRNAs on 24 HPVs of unambiguous subtypes of risk. The site is targeted by two less common human miRNAs, miR-875 and miR-3144, and is located in E6 oncogene open reading frame (ORF) and overlap with the first alternative splice exon of viral early transcripts. In validation tests, miR-875 and miR-3144 were identified to suppress the target reporter activity markedly and inhibit the expression of both synthetically exogenous E6 and endogenous E6 oncogene. High level of two miRNAs can inhibit cell growth and promote apoptosis in HPV16-positive cervical cancer cells. This study provides a promising common target of miRNAs for most HR-HPVs and highlights the effects of two low expressed human miRNAs on tumour suppression.

## INTRODUCTION

MicroRNAs (miRNAs) are small non-coding RNAs that modulate gene expression at post-transcriptional level for degradation of the target mRNAs or inhibiting translation of the encoded protein, playing critical roles in a wide spectrum of biological processes (Ambros, 2004). They bind to complementary target sites in mRNA that are often located in 3′ untranslated region (UTR) but can also be found in 5′UTR or even coding region (Forman and Coller, 2010; Li et al., 2011). Recent data provide increasing evidence that human miRNAs may directly affect viral gene expression contributing to host’s innate anti-viral defense or viral immune escape (Ghosh et al., 2009; Mahajan et al., 2009). In 2005, one human miRNA (has-miR-32) was reported to be able to target sequence in primate foamy virus type 1 (PFV-1) genome thereby inhibiting viral replication and translation (Lecellier et al., 2005). Currently, more and more human miRNAs that target hepatitis C virus (HCV), hepatitis B virus (HBV), human immunodeficiency virus (HIV), influenza A Virus H5N1 and H1N1 were found (Houzet et al., 2012; Pedersen et al., 2007; Potenza et al., 2011; Scaria et al., 2006; Song et al., 2010). It is noted that many of these target sequences, such as the target of miR-125 in HBV, the targets of miR-448 and miR-122 in HCV (Li et al., 2011; Pedersen et al., 2007; Potenza et al., 2011), were found well conserved through viral genomes, and several of them have been preferentially exploited for new anti-viral strategies (Russo and Potenza, 2011).

Human papillomaviruses (HPVs) are non-enveloped, double-stranded circular DNA viruses and are associated with a wide spectrum of benign and malignant neoplasia (Zheng and Baker, 2006). Until now, more than 120 HPV subtypes have been identified and characterized (Bernard et al., 2010). According to oncogenic potentials epidemiologically, some of them are classified into “high-risk (HR)” group and “low-risk (LR)” group (Munoz et al., 2003). Persistent infection of multiple HR-HPVs is the major cause of cervical cancer in female. As a representative HR-HPV, HPV16 is responsible for approximately ~60% of invasive cervical cancers worldwide (Zheng and Baker, 2006). HR-HPVs bound up with malignancies have developed many oncogenic strategies which are rarely found in LR-HPVs. Examples include that viral crucial oncoproteins efficiently degrade tumor suppressors (such as E6 target p53) (Scheffner et al., 1990), and integration of the viral DNA segments into the human genome increases E6 expression leading to oncogenesis (Alp Avci, 2012).

While the role of miRNAs in cervical cancer, as well as in other cancers, has been extensively studied during the past few years (Hayes et al., 2014; Jimenez-Wences et al., 2014), few miRNAs have been reported to be involved in the regulation of HPV viral life cycle, and those researches were based on limited HR-HPV subtypes (Gunasekharan and Laimins, 2013; Jung et al., 2014; Melar-New and Laimins, 2010; Nuovo et al., 2010). Since co-infection of multiple HR-HPVs is common in cervical cancer (Mejlhede et al., 2010), we wondered that whether there are some human miRNAs who can target the majority of HR-HPVs. Our previous study found that HPVs in different group with specific oncogenic potential (HR or LR group) have distinguishable divergence on phylogenetic genotypes (Li et al., 2009). We also noticed that miRNAs are conserved, and are promising phylogenetic markers for interpreting evolution of organisms (Qingqing Cai, 2010). Therefore, it seems possible to find some candidate miRNAs and conserved target sites distinguishable between HR-HPVs and LR-HPVs.

In this study, we employed bioinformatics approaches to look for potential conserved target among the majority of HR-HPV genomes for human miRNAs and investigate the target effects of candidate miRNAs on HPV oncogene and cervical cancer cells. It might provide a promising common target of miRNAs for most HR-HPVs and focus on the effects of two low expressed human miRNAs on tumour suppression.

## RESULTS

### *In silico* analysis yielded most promising target sites of human miRNAs in HPV genome

**Figure 1:**
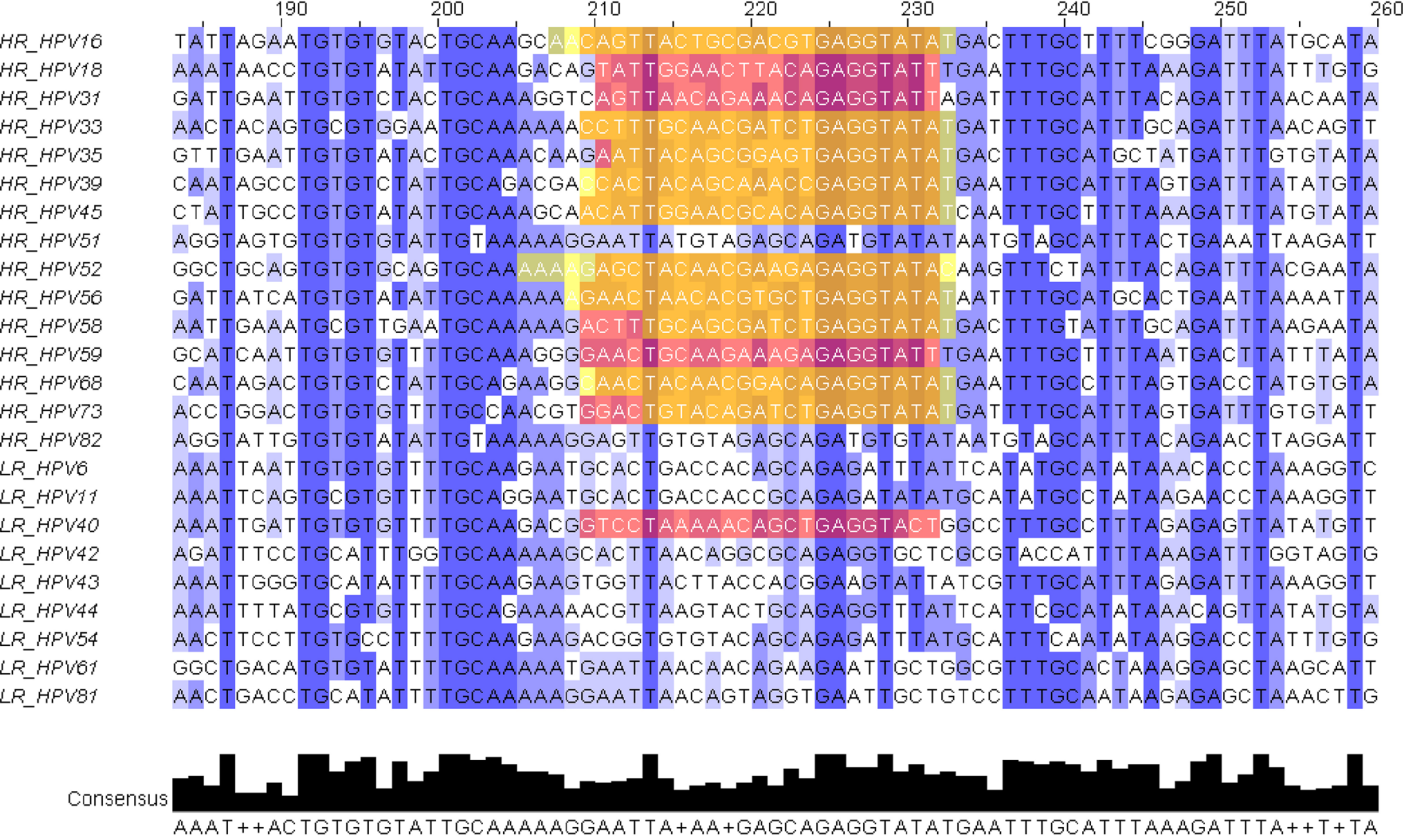
**The sequence alignments for 15 HR-HPV subtypes (HPV16, 18, 31, 33, 35, 39, 45, 51, 52, 56, 58, 59, 68, 73, 82) and 9 LR-HPV subtypes (HPV6, 11, 40, 42, 43, 44, 54, 61, 81)**. Yellow highlights the targets of miR-875, red highlights the targets of miR-3144, orange show their overlapping districts. Blue shows the degree of conservation in each Nucleotide site. The more conserved it is, the darker blue it shows. Black columns at the bottom show the consensus of conservation in each Nucleotide site

15 HR-HPV subtypes (HPV16, 18, 31, 33, 35, 39, 45, 51, 52, 56, 58, 59, 68, 73, and 82) and 9 LR-HPV subtypes (HPV6, 11, 40, 42, 43, 44, 54, 61, and 81) were obtained from PaVE: Papilloma virus genome database (http://pave.niaid.nih.gov/). Sequences were allocated according to genes and regions annotation and formed 8 representing regions including E1, E2, E4, E6, E7, L1, L2, and URR which existed in almost all types of HPVs. The sequences of each gene or region were aligned by MUSCLE with default parameters. All sequences were subjected to scanning for the putative binding sites of 2042 human mature miRNAs by miRanda. Firstly, we calculated the presumed binding efficiency between HR-HPV group and LR-HPV group of each gene (or URR) region roughly: If a miRNA has (at least) one binding site on a gene part in one HPV subtype, we noted the binding situation as 1 (existing), otherwise we noted as 0 (none). Then binding efficiency was summarized according to HPV risk groups. For example, miR-4282 was predicted to bind L1 genes of 15 HPV subtypes. Then the binding efficiency of miR-4282 and L1 were recorded as 15 (13 for HR-HPVs, while 2 for LR-HPVs). Top 12 miRNAs who demonstrated a strong preference for either risk group (the utmost absolute difference between HR-HPVs and LR-HPVs) was listed in Table 1. In order to study the binding pattern, the binding sites of 12 selected miRNAs were mapped to HPV alignments. Amazingly, most of the binding sites were randomly located among HPVs or formed small conservation sites, while the potential targets of miR-875 and miR-3144 located in E6 oncogene ORF happened to form a conserved target site almost unique in HR-HPVs as was shown in Fig. 1. For HPV16, miR-875 was supposed to target segment nt 209–231 and miR-3144 was supposed to target segment nt 207–232. The detailed sequences were showed in Table 2.

**Table 1:** **Most differentiated binding situation between HR-HPV group and LR-HPV group**

Rank	Gene part	MiRNA	Binding efficiency of 24 HPVs	Binding efficiency of HR-HPVs	Binding efficiency of LR-HPVs	Absolute difference
1	E6	MiR-875	13	13	0	13
2	E6	MiR-3144	12	12	0	12
3	L1	MiR-4282	15	13	2	11
4	E1	MiR-2355	13	12	1	11
5	E1	MiR-365	11	11	0	11
6	L2	MiR-889	20	15	5	10
7	E1	MiR-626	18	14	4	10
8	E2	MiR-302a	14	12	2	10
9	E2	MiR-2355	14	12	2	10
10	E1	MiR-26b	12	11	1	10
11	L2	MiR-3665	12	11	1	10
12	L1	MiR-1299	10	10	0	10

**Table 2:** **Sequences and names of constructed plasmids**

Name	Sequences	Vector	Constructed plasmids
**MiR-875**	UAUACCUCAGUUUUAUCAGGUG	pSilencer4.1-CMV	MiR-875 Expression vector
Target: 209–231 nt*	CAGTTACTGCGACGTGAGGTATA	psiCheck-2	209–231 Luciferase reporter
Mutated target	CAGTTACTGCGACGTGAGGATTA	psiCheck-2	∆209–231 Mutated reporter
**MiR-3144**	AUAUACCUGUUCGGUCUCUUUA	pSilencer4.1-CMV	MiR-3144 Expression vector
Target: 207–232 nt*	AACAGTTACTGCGACGTGAGGTATAT	psiCheck-2	207–232 Luciferase reporter
Mutated target	AACAGTTACTGCGACGTGAGGATTAT	psiCheck-2	∆207–232 Mutated reporter
**E6**	NC_001526, nt 83–559 (498 bp)**	pcDNA3.1	pcDNA3.1-E6

* Sites in HPV16 genome

** Sequence from HPV16 genome (NC_001526)

### MiR-875 and miR-3144 target the HPV genomic sites in luciferase reporter

**Figure 2:**
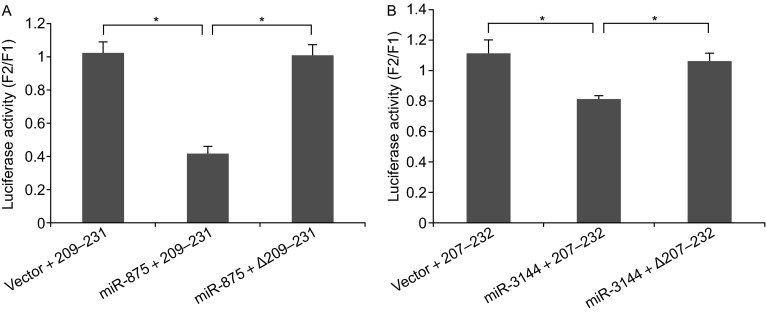
**MiR-875 and miR-3144 inhibit luciferase reporter expression**. (A) Analysis of the relative luciferase activity in C33A cells co-transfected with miR-875 vector and target (209–231) reporter, or empty vector and target (209–231) reporter, or miR-875 vector and mutated target (∆209–231) reporter. (B) Analysis of the relative luciferase activity in C33A cells co-transfected with miR-3144 vector and target (207–232) reporter, or empty vector and target (207–232) reporter, or miR-3144 vector and mutated target (∆207–232) reporter. *P*-values at Student’s *t*-test were *P < 0.05

As bioinformatics analysis yielded the most promising target sites of human miRNAs in HPV genome, it was important to determine if the seed sequences actually functioned as target for these miRNAs. The most common and direct methods for miRNA target validation are based on fluorescent reporter gene constructs transfected in cultured cells (Kuhn et al., 2008). The HPV genomic segments containing putative target sites for miR-875 and miR-3144 were then individually cloned downstream of the Renilla luciferase open reading frame contained in the psiCheck-2 vector as reporter. These luciferase reporters were subsequently transfected into C33A cells along with miRNA expression vectors and assayed for luciferase expression. The results obtained from two independent experiments, expressed as Renilla luciferase activity relative to that of control firefly luciferase, are shown in Fig. 2. In C33A cells, co-transfection with miR-875 and its target (209–231) decreased the relative luciferase activity to 49% compared with empty pSilencer4.1-CMV vector and target, and co-transfection with miR-3144 and its target (207–232) decreased the activity to 71% compared to empty vector and target. To examine whether the interaction between miRNAs and their target sequences direct or indirect, we mutated the miRNA binding sites with one or two nucleotide mutations (Table 2). As a result, with those mutation presented in reporter (∆209–231, ∆207–232), luciferase expression failed to be regulated by miR-875 and miR-3144 (Fig. 2). These experiments demonstrated that miR-875 and miR-3144 interacted directly with their binding sites to regulate luciferase reporter expression, and the effect was supposed to be highly specific, as it was abolished when the target sequence was single-nucleotide or two-nucleotide mutated.

### MiR-875 and miR-3144 negatively regulate exogenous E6 gene expression

**Figure 3:**
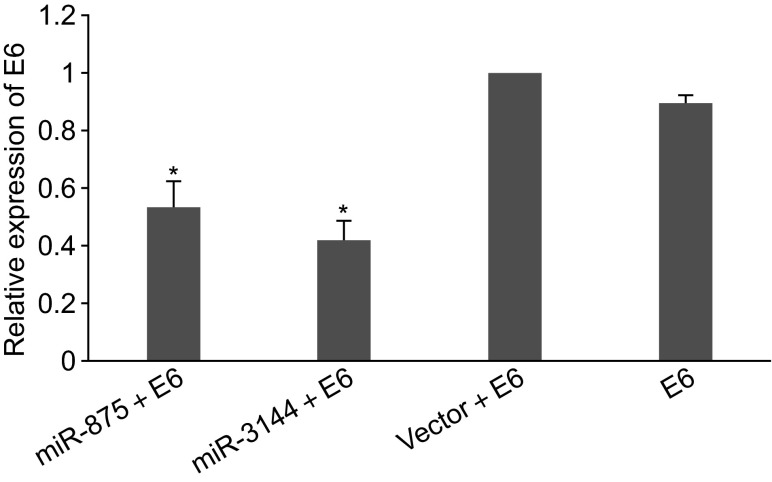
**MiR-875 and miR-3144 decrease the expression of exogenous E6**. The relative expression of E6 mRNA obtained by qRT-PCR in C33A cells after co-transfection with synthetically E6 construct along with miRNAs vectors or empty vector. *P*-values at Student’s *t*-test were *P < 0.05

MiRNAs can suppress the expression of target genes through translational repression or through degradation of a target’s transcript. Since miR-875 and miR-3144 can down-regulate the luciferase expression by binding to their target in E6, we investigated whether the two miRNAs could directly regulate E6 expression. We transfected synthetically E6 construct (pcDNA3.1-E6) into HPV-negative cervical carcinoma cell lines (C33A). Although E6 gene is transcribed through bicistronic E6/E7 mRNA, studies show that transfection with monocistronic E6 into C33A cells could also result in a stable expression of unspliced E6 mRNA (del Moral-Hernandez et al., 2010). Some of these cells were then transfected with miRNAs expression vectors or empty pSilencer4.1-CMV vector. Then quantitative real-time RT-PCR was used to quantitate the E6 mRNA level in all group cells. The results obtained from this experiment, expressed as relative expression normalized with internal control gene homo-Actin. As shown in Fig. 3, transfection with synthetically E6 construct (E6) or along with empty pSilencer4.1-CMV vector (vector + E6) result in stable expression of E6 mRNA, while transfection with E6 construct along with miR-875 (miR-875 + E6) or miR-3144 (miR-875 + E6) individually decreased the expression of E6 mRNA to 53% or 42%.

### MiR-875 and miR-3144 negatively regulate endogenous E6 gene expression

**Figure 4:**
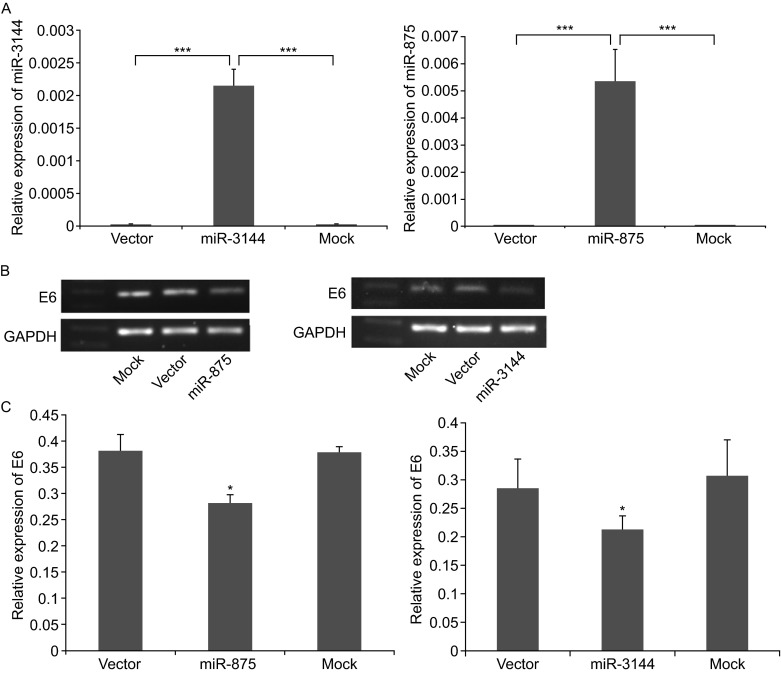
**Transfection with miR-875 and miR-3144 interferes the expression of endogenous E6**. (A) The expression levels of miR-875 or miR-3144 by qRT-PCR in SiHa cells transfected with miR-875 or miR-3144 expression vectors, SiHa cells transfected with empty vectors (Vector), or untreated SiHa cells (Mock). (B and C) RT-PCR analysis of E6 mRNA relative expression in SiHa cells transfected with miR-875 or miR-3144 expression vectors, SiHa cells transfected with empty vectors (Vector), or untreated SiHa cells (Mock). *P*-values at Student’s *t*-test were *P < 0.05, ***P < 0.001

The experimental model above was used to measure the repression effect on exogenous E6, we further investigated whether miR-875 and miR-3144 could interfere with the expression of endogenous E6 oncogene. MiR-875 and miR-3144 expression vectors were then transfected into cervical cancer SiHa cells which contain integrated HPV16 DNA and have detectable level of E6 oncogene mRNA (Rosenberger et al., 2010). Empty pSilencer4.1-CMV vector (Vector) and untreated SiHa cells (Mock) were used for controls. As shown in Fig. 4A, the expression levels of miR-875 and miR-3144 in SiHa cells after transfection obtained by RT-qPCR were significantly increased compared to controls. The RT-PCR was then used to detect the average cellular content of E6 oncogene mRNA. As shown in Fig. 4B and 4C, transfection with miR-875 decreases the cellular content of E6 mRNA by 26.0% compared to Mock control and 26.4% to Vector control. Likewise, transfection with miR-3144 decreases the E6 mRNA by 30.5% compared to Mock control and 25.3% to Vector control. The regulation was believed to be remarkable if we consider the possible pre-existing expression of other endogenous targets of the two miRNAs, as one miRNA always target more than one gene. Taken together, these results indicated that miR-875 and miR-3144 negatively regulate not only exogenous E6 gene expression from synthetic construct but also endogenous E6 oncogene expression in an *in vitro* model through mRNA degradation.

### High level of miR-875 and miR-3144 inhibit cell growth in SiHa cells

**Figure 5:**
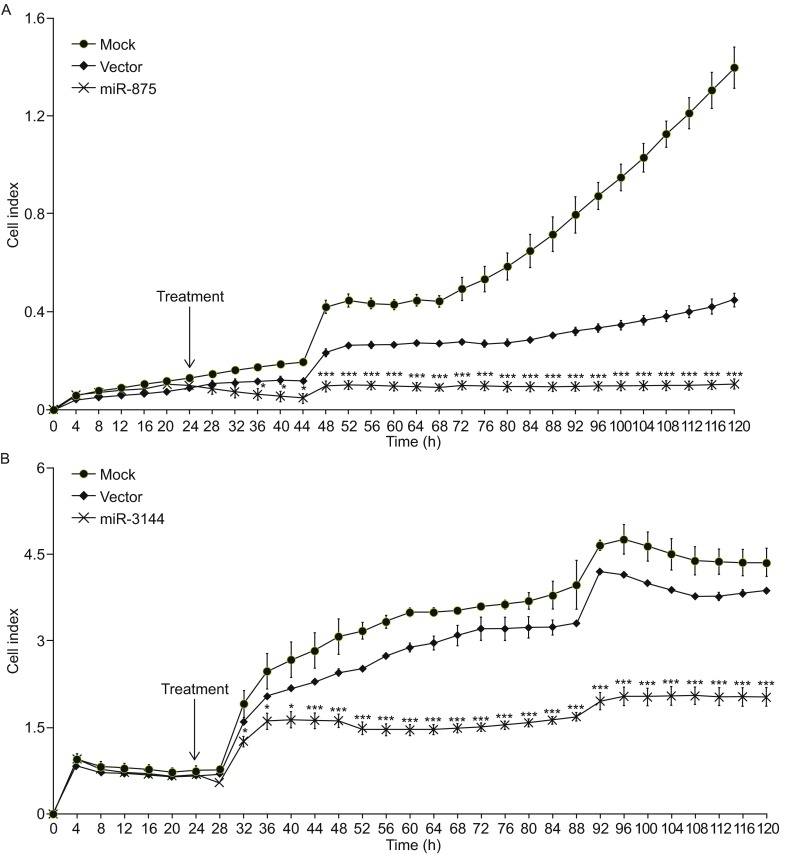
**High-level of miR-875 and miR-3144 inhibit cell growth**. Time kinetics of cell growth after 120 h of monitoring every 4 h with the xCELLigence RTCA DP System in SiHa cells transfected with miR-875 (A) or miR-3144 (B) vectors, or with empty vectors (Vector), or untreated SiHa cells (Mock). *P*-values at Student’s *t*-test were *P < 0.05, ***P < 0.001

In order to detect the effect of increased level of miR-875 or miR-3144 on cancer cell growth, miR-875 and miR-3144 expression vectors were individually transfected into SiHa cells and the increased levels of the two miRNAs were confirmed by RT-qPCR as described previously. Untreated cells (Mock) and cells transfected with empty vector (Vector) were used for controls. The xCELLigence RTCA System was then performed to monitor the proliferative potential of those cells. In this system, the growth rate of cells was measured by monitoring the cell index which depend on the electronic readout of cell sensor impedance with real-time and continuous record every 4 h. After 120 h of monitoring cell proliferation with this System, we observed a continuous decrease of cell growth and motility in both miR-875 transfected cells and miR-3144 transfected cells. Instead, those cells in two control groups continued to grow smoothly during this period (Fig. 5), suggesting that overexpression of miR-875 and miR-3144 could inhibit the growth of cervical cancer cells.

### High level of miR-875 and miR-3144 induces apoptosis in SiHa cells

**Figure 6:**
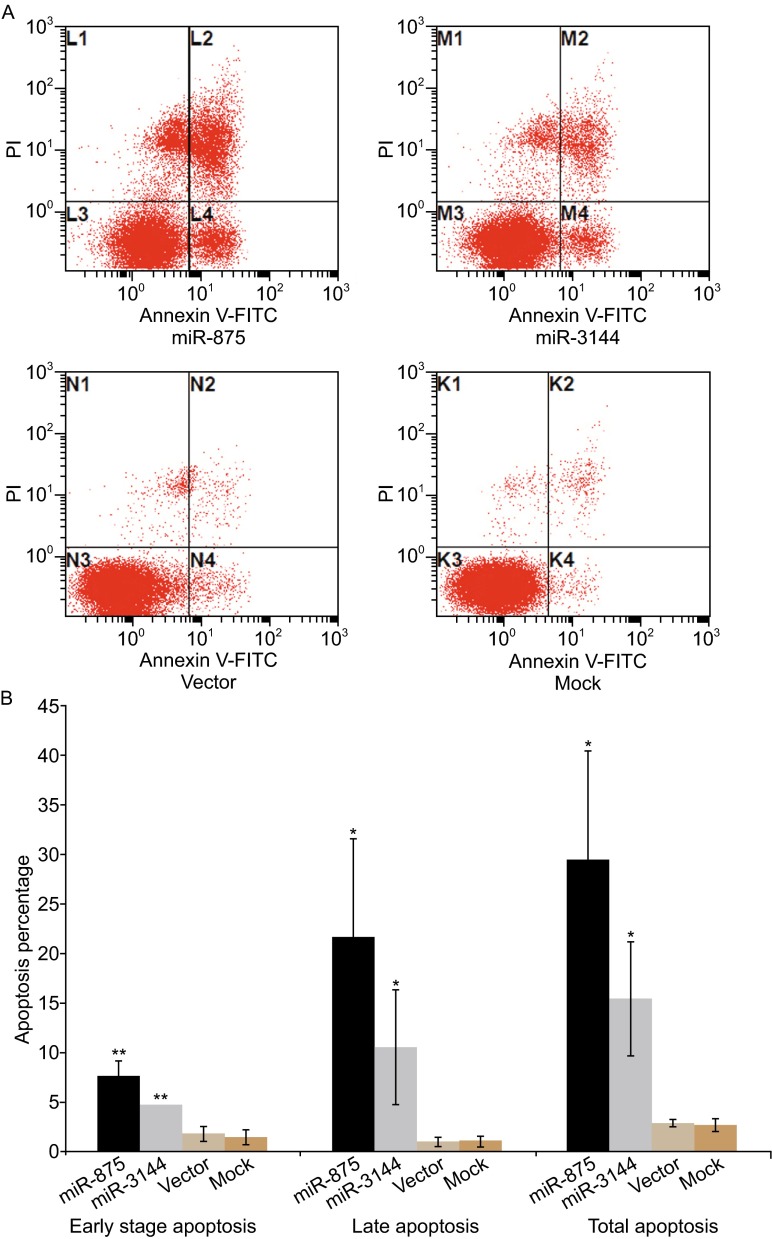
**High-level of miR-875 and miR-3144 promoted cell apoptosis**. (A) Annexin-V/PI analysis in SiHa cells transfected with miR-875 or miR-3144 vector, or with empty vectors (Vector), or untreated SiHa cells (Mock). (B) Early apoptosis, late apoptosis and the sum total of apoptosis in different group cells. *P*-values at Student’s *t*-test were *P < 0.05, **P < 0.01

In order to determine whether increased level of miR-875 or miR-3144 induce apoptosis of cervical cancer cell, flow cytometric analysis of cell apoptosis was performed for SiHa cells transfected with miR-875 or miR-3144 expression vectors, SiHa cells transfected with empty vectors (Vector) and untreated SiHa cells (Mock). As shown in Fig. 6A, SiHa cells in two control groups were primarily Annexin V/propidium iodide (PI) negative and remained viable, but the SiHa cells with transfection of miR-875 and miR-3144 vectors were observed to undergo apoptosis in both early and late periods, which were shown by Annexin-V+/PI- for early apoptotic stage and annexin-V+/PI + for late apoptotic stage. And the rate of early apoptosis, late apoptosis and total apoptosis were calculated and shown in Fig. 6B. The results showed that overexpression of miR-875 and miR-3144 can significantly increase apoptosis in HPV-positive cervical cancer cells.

**Figure 7:**
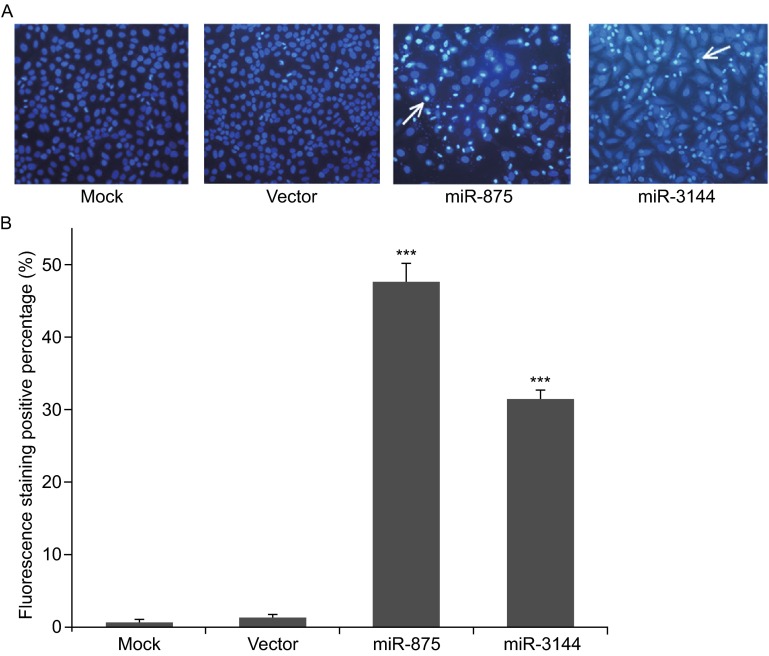
**High-level of miR-875 and miR-3144 induced apoptotic morphological changes**. (A) Apoptotic morphological changes (white arrows) with Hoechst staining (blue) in fluorescence microscope (200 × times) in SiHa cells transfected with miR-875 or miR-3144 vector, or with empty vectors (Vector), or untreated SiHa cells (Mock). (B) Histograms for quantification of the cell death (%) in different groups. *P*-values at Student’s *t*-test were ***P < 0.001

The effects of overexpression of miR-875 and miR-3144 on nuclear morphology were measured by Hoechst 33258 staining, since the morphological changes in the nuclear chromatin is indicative of the apoptotic process. As shown in Fig. 7A, SiHa cells with transfection of miR-875 and miR-3144 vectors exhibited reduced nuclear size, intense fluorescence, chromatin condensation, nuclear fragmentation, and presence of apoptotic bodies evident and were considered apoptotic cells. As shown in Fig. 7B, the percentage of cells with apoptotic morphology was significantly increased in with miR-875 and miR-3144 transfected cells, compared with cells in two control groups.

## DISCUSSION

Cervical cancer, which is caused by persistent infection with high-risk HPVs, is the second most common type of cancer in women worldwide (Stewart and Wild, 2014). While recent reports have highlighted the role of miRNAs as critical effectors in intricate host-virus interaction networks, we address whether human miRNAs influence HPV oncogene in this study. We employed bioinformatics approaches scanning all potential targets of human miRNAs on 24 unambiguous risk-type HPVs. A well-conserved target site among most HR-HPVs of two candidate miRNAs, miR-875 and miR-3144, has been found. By using the luciferase reporter, synthetically constructs and cultured cells, we show that miR-875 and miR-3144 can cause silencing effect of target reporter and interfere with the expression of E6 oncogene. In the presence of high-level miR-875 and miR-3144, HPV-positive cervical cancer cells undergo apparent growth retardation and apoptosis promotion.

The targets of miR-875 and miR-3144 were found located in encoding region of E6, which is the main oncogene associated with oncogenicity of HR-HPVs. E6 oncoprotein was known to be able to directly target and degrade the tumor suppressor p53, therefore inhibit p53-mediated apoptosis, reduce p21-mediated cell cycle regulation, and destabilize chromosomes, favoring tumor progression of cervical cancer (Hausen, 2002). In this study, we demonstrated that miR-875 and miR-3144 could suppress the expression of both exogenous E6 and endogenous E6 by directly targeting E6 ORF. It should be noted that the first alternative exon of HR-HPVs’ early transcripts in E6 ORF region can be alternatively spliced and lead to at least four species of mRNA transcripts with various coding potential in HPV16 (5′ splice site at 226 nt and three alternative 3′ splice site at 409 nt, 526 nt, or 742 nt) (Zheng and Baker, 2006). Among them, only un-spliced E6 full-length transcript encodes E6 oncoprotein and exhibit transforming capacity, whereas other spliced transcripts E6* counteract E6 oncoprotein functions (Filippova et al., 2007; Pim and Banks, 1999). The target sites of miR-875 and miR-3144 in this study were found overlap to this alternative splice exon and thereby target only full-length transcript of E6 but no other spliced transcripts. Since the primers used for PCR in this study cover the 5′ splice site which only exists in un-spliced transcript of E6, our data support that miR-875 and miR-3144 interfere with expression of E6 full-length transcript, the latter was considered as the only source of the E6 oncoprotein (Stacey et al., 1995). Interestingly, alternatively spliced E6 transcripts have so far only been described for HR-HPVs but not for LR-HPVs (Hernandez-Lopez and Graham, 2012), implying that this region may have functional relevance on biological processes of HR-HPV carcinogenesis. Since we have little knowledge on the regulation of HPV at the level of RNA splicing (Zheng and Baker, 2006), whether the reason why the targets of miR-875 and miR-3144 precisely overlap to this region is a coincidence or that they are involved in regulation of alternative splicing is not yet known.

So far, little is known about the biological function of miR-875 (located in Chr 8q22.2) and miR-3144 (located in Chr 6q22.31, a common fragile site in which HPV16 integration happens frequently) (Thorland et al., 2000; Zhao et al., 2012), which were first reported publicly in 2007 and 2010 respectively (Landgraf et al., 2007; Stark et al., 2010). Both of them have previously been reported by Daniela Witten and his colleagues to be expressed at rather low levels in human cervical tumor tissues, including squamous cell carcinoma tissue and adenosquamous cell carcinoma tissue, with median expression 1.5 reads observed for miR-875 and 1 reads observed for miR-3144 (Witten et al., 2010). Based on our observations, it is reasonable to explain that a suitable low level of miRNAs with antiviral effect is beneficial for HPV’s survival and persistent latency favoring carcinogenesis. Moreover, miR-875 and miR-3144 have also been reported to be dysregulated in several types of cancer, such as pancreatic cancer, clear cell renal cell cancer, prostate cancer, colorectal cancer, and upper tract urothelial cancer (Hamfjord et al., 2012; Hao et al., 2011; Nguyen et al., 2013; Weng et al., 2010; Zaravinos et al., 2014). Similar to the present study, miR-875 was considered as one of the 14 candidate miRNAs which potentially target EGFR and act as the therapeutic target of lung cancer in Lawrence WC Chan’s study (Chan et al., 2012), and miR-875 was also linked to cell apoptosis pathways in dopaminergic neuronal cells and severe H1N1 viral infected cells (Juan Moran, 2014; Li et al., 2013). Taken together, although only a few studies have been done on the function of miR-875 and miR-3144, evidence regarding miR-875 or miR-3144 mediated tumor suppression is beginning to emerge.

However, the biological role of this effect on natural HPV carcinogenesis remains unclear. It may be insufficient to eliminate the viruses but only counteract viral replication to evade immune elimination and establish a persistent infection with a long host survival and a high spread of the virus in the human population, like HBV, HCV, and HIV-1 (Russo and Potenza, 2011). Therefore, forced high level of miR-875 and miR-3144 in HPV-positive cervical cancer cells as we show in this study could unbalance the HPV-host coexistence and may re-direct the outcome from a persistent HR-HPV infection to a complete recovery.

This study adds to the growing body of evidence implicating the association between miRNA and HPV in carcinogenesis. Aberrant cellular miRNAs expression profiles in HPVs infected cervical cancer tissue or cell have been reported (de Freitas et al., 2014). Several host miRNAs can be modulated by oncogenic HPVs (Greco et al., 2011; Martinez et al., 2008; Shi et al., 2012; Zheng and Wang, 2011). HPVs were found to encode their own miRNA species lately (Qian et al., 2013). However, limited investigations refer to the interplay between HPV genes and miRNAs. Until recently, some miRNAs were suggested to be involved in HPV regulation which were based on limited HPV subtypes, shown by that miR-203 have inhibitory effect on HPV16 DNA replication, miR-125b and miR-145 suppress HPV31 amplification, and miR-375 interact with E1 and E7 gene of HPV16 (Gunasekharan and Laimins, 2013; Jung et al., 2014; Melar-New and Laimins, 2010; Nuovo et al., 2010). This work provides two less common human miRNAs who can target HPV16 E6 oncogene which the target site is a promising common target for most HR-HPVs. So far, therapeutics for clearing persistent HR-HPV infection are still very limited (Harper and Demars, 2014). The optimal anti-HPV strategy should be against multiple HR-HPV subtypes, since co-infection is rather common in cervical cancer (Mejlhede et al., 2010). Although this study is preliminary with only *in vitro* experiments, we believe the results add to the novel knowledge for anti-HPV strategies.

While the prevailing wisdom is that miRNAs bind to the 3′UTR of target genes, this study shows that miR-875 and miR-3144 bind to the coding region of E6. The possible explanation is that HPV genome is limited and 3′UTR sequence is not long enough. Likewise, evidence in some other viruses, like HBV and HCV, reveals that coding region or 5′UTR could be targeted by miRNAs (Li et al., 2011; Potenza et al., 2011). Moreover, our result is consistent with Anita Dreher’s assumption that 3′UTR in HPV genome might be not a target for miRNA regulation (Dreher et al., 2011). Zheng et al. have hypothesized that several dozen potential miRNA binding sites exist in the genome-wide distribution of HPV (Harper and Demars, 2014), suggesting a need for further investigation to unveil more cellular miRNA-mediated regulation of HPV.

## MATERIALS AND METHODS

### Materials

The psiCheck-2 vector was purchased from Promega. The pSilencer4.1-CMV vector and pcDNA3.1 vector were obtained from Ambion. DNA oligonucleotides were obtained from Invitrogen. The HPV 16-positive SiHa cervical cancer cell line and HPV-negative C33A cervical cancer cell line were used in this study.

### Computational analyses

The standard viral genome sequences were obtained from PaVE: Papilloma virus genome database (http://pave.niaid.nih.gov/). All human mature miRNA sequences were obtained from miRBase Release 14.0 (http://www.mirbase.org/) (Griffiths-Jones et al., 2008). Alignment of the DNA sequences of the viral genomes was performed with MUSCLE 3.6. The miRanda-3.3a was used to scan the HPV genomic sequence for the potential target sites for miRNAs (John et al., 2004). The modes of hybridization in computer programs were based on the principles of miRNA-target recognition containing the following parameters: complementarity on local base pairs, RNA-RNA double chain free energy, hybridization temperature, conservation of the site, and miRanda score (Bentwich, 2005).

### Plasmid constructs

Nucleotide sequences of putative miRNAs binding sites in HPV were obtained by chemical synthesis of complementary oligonucleotides and were cloned into psiCheck-2 vectors at *Xho*I and *Not*I sites as luciferase reporter. The mutated target luciferase reporter bearing single-nucleotide or two-nucleotide mutated target sequences were obtained by the same approach. Human miRNA precursors were cloned into pSilencer4.1-CMV vectors at *Bam*HI and *Hin*dIII sites to construct miRNA expression vectors. A 498 bp fragment containing full-length E6 ORF of HPV16 (NC_001526, nt 83–559) were amplified by PCR and cloned into pcDNA3.1 vector at *Nhe*I/*Xho*I sites, resulting in E6 construct named pcDNA3.1-E6. All the selected constructs were then sequenced to confirm their identity. Sequences and the names of constructed plasmids were showed as detailed in Table 2.

### Cell cultures and transfections

SiHa cells and C-33A cells were cultured in DMEM with 10% fetal bovine serum at 37°C and 5% CO_2_. Cells were washed with PBS and switched to antibiotic-free growth medium for 24–48 h before transfection. Transfections were performed with cells at 80%–90% of confluence by using of Lipofectamine 2000 (Invitrogen) according to the manufacturer’s protocol.

To determine whether miRNAs could bind to their putative target sites in luciferase reporter, C33A cells were co-transfected with target luciferase reporter or mutated target reporter, and with matched miRNAs expression vector. The empty pSilencer4.1 vector was used as a negative control. After 24 h, the cells were harvested for relative luciferase assay analysis.

To determine the effects of miRNAs on E6 expression from exogenous plasmids, C33A cells were co-transfected with pcDNA3.1-E6 vector and miRNAs expression vector. The empty pSilencer4.1 vector was used as control. After 48 h, the cells were collected and analyzed for E6 mRNA level by RT-PCR.

To determine the effects of miRNAs on endogenous E6 expression, HPV16-infected cervical cancer SiHa cells were transfected with miRNAs expression vector or empty pSilencer4.1 vector. Untreated SiHa cells were used as another control. After 48 h, the cells were collected for RT-PCR analysis.

In order to determine whether miRNAs influence the proliferation and apoptosis of HPV infected cancer cells, SiHa cells were transfected with miRNAs expression vector or empty pSilencer4.1 vector. Untreated SiHa cells were used as another control. The cells were collected for Real time cell analysis (RTCA), Annexin V assay and Hoechst staining assay.

### Luciferase report assays

Luciferase assays were performed 24 h after transfection of C33A cells using the Dual-Luciferase Reporter Assay System (Promega) following the manufacturer’s instructions. The mean of the luciferase activities measured with Luminoskan TL Plus luminometer (Thermo Labsystems) were used to calculate ratios between Renilla lucsiferase (F2) and firefly luciferases (F1).

### Quantitative real-time RT-PCR

Total RNA was extracted from cultured cells using Trizol Reagent (Invitrogen) following the manufacturer’s instructions. For miRNA analysis, mature miRNAs were reverse transcribed from total RNA using specific stem-loop RT primers. Per 15 µL RT reaction included: 5× M-MLV buffer, dNTP, M-MLV (200 U), RNase inhibitor (40, specific stem–loop RT primers (5 μmol/L), total RNA (1 µg) and H_2_O. The mix then incubated at 16°C for 30 min, 42°C for 30 min, 85°C for 5 min, at last the product chilled on ice for 5 min.

For E6 analysis, 1 μg RNA was diluted in H_2_O along with 1 µL of Oligo dT Primer (Invitrogen) to obtain a total volume of 12 μL. Samples were incubated at 65°C for 5 min and chilled on ice for 3 min. Then, 1 μL of RNaseout (Invitrogen), 2 μL 0.1 mol/L dTT (Invitrogen), and 4 μL 5× First Strand Buffer (Invitrogen) were added and samples were then incubated at 25°C for 10 min.

Real-time PCR apparatus (CFX96, Bio-Rad), associated with CFX Manager Software (version 1.6, Bio-Rad), was used for the real-time PCR. Each reaction was done in duplicate in a volume of 20 µL with 96-well optical-grade PCR plates, sealed with optical sealing tape (Bio-Rad). Amplification reactions were done with SYBR Green Supermix (Bio-Rad) and following temperature profiles: one cycle at 95°C (3 min), 40 cycles of denaturation at 95°C (30 s), annealing at 60°C (30 s), extension at 72°C (30 s) and one final cycle at 95°C (30 s). Melt curve analyses were performed by slowly heating the PCR mixtures from 65°C to 95°C (1°C per cycle of 10 s) with simultaneous measurements of the SYBR Green I signal intensities. Relative expressions were calculated by the formula 2^−∆∆Ct^.

For semi-quantitative PCR, cDNA was diluted 1:40 in H_2_O and 1 μL were amplified in 20 μL with 2× PCR Master Mix using 1 μL of a 10 μmol/L primer mix. The PCR reaction was conducted at 95°C for 3 min, followed by 30 cycles of 95°C for 10 s, 60°C for 30 s and 72°C for 1 min. The intensities of products obtained were measured by TotalLab100 software. Primers used in this study were shown in Table 3.

**Table 3:** **Primers used in this study**

Target	Primer name	Primer sequence (5′ to 3′)	Application
E6	E6-F	AGCGACCCAGAAAGTTACCA	QPCR
	E6-R	GCATAAATCCCGAAAAGCAA	
E6	Q-E6-F2 (QP357)	AGCGACCCAGAAAGTTACCA	RT-PCR
	Q-E6-R2 (QP358)	AATTCCTAGTGTGCCCATTAAC	
MiR-3144	Q-miR-3144-RT	CTCAACTGGTGTCGTGGAGTCGGCAA TTCAGTTGAGCTATATAT	RT
	Q-miR-3144-F	ACACTCCAGCTGGGAGGGGACCAAAGAGAT	QPCR
MiR-875	Q-miR-875-RT	CTCAACTGGTGTCGTGGAGTCGGCAATTCA GTTGAGCACCTGAT	RT
	Q-miR-875-F	ACACTCCAGCTGGGTATACCTCAGTTTTAT	QPCR
U6	U6-RT	CGCTTCACGAATTTGCGTGTCAT	RT
	U6-F	GCTTCGGCAGCACATATACTAAAAT	QPCR
Downstream universal primer	MiR-R	CGCTTCACGAATTTGCGTGTCAT	QPCR

### Real time cell analysis (RTCA)

In order to measure the proliferative potential of cells, SiHa cells were seeded in a density of 1–2 × 10^5^ cells/well in E-plate according to the manufacturer’s instructions and were monitored every 4 h using a real time cell analyzer (RTCA, xCELLigence, Roche). In this system, cells adhere to the bottom of each well, covering the surface of the sensor that monitors cells by measuring their cell index that is translated from the electronic readout. The cell growth curves were recorded for 120 h to assess the capability of cell proliferation.

### Flow cytometric analysis of cell apoptosis

In order to quantify early apoptosis and late apoptosis induced by miRNAs, cell apoptosis was measured using the Annexin V Apoptosis Detection Kit according to the manufacturer’s protocol. Briefly, at 24 h after transfection with vectors, SiHa cells were trypsinized and washed with cold PBS, and then resuspended in 100 μL 1× binding buffer at 1 × 10^6^ cells/mL. 100 μL of cells were then added with 5 μL of Annexin V and 1 μL of propidium iodide (PI) and were incubated for 15 min at room temperature in the dark. After incubation, 400 μL 1× binding buffer was added to each tube, and the samples were analyzed by flow cytometry (Beckman-Coulter) within 1 h.

### Hoechst staining assay

In order to examine the nuclear morphological changes in cells, cells were stained with Hoechst 33258, and visualized by fluorescence microscope according to the manufacturer’s protocol. Briefly, SiHa cells were plated with 2.5 × 10^6^ cells/mL in 6-well plates. At 24 h after transfection, cells were then collected by centrifugation, washed with phosphate buffered saline (PBS), fixed with paraformaldehyde and stained with 0.5 mL (10 μg/mL) Hoechst 33258 in darkness for 5 min at room temperature. The results were observed and recorded under a fluorescence microscope. These cells exhibited reduced nuclear size, intense fluorescence, chromatin condensation, and nuclear fragmentation, were considered apoptotic cells. The percentage of Hoechst-positive apoptotic cells per visual field was determined.

### Statistical analysis

All experiments were performed in triplicate in three independent experiments. The data were tested for significance employing the *t*-test and presented as mean ± SD. The level of significance was set at *P* < 0.05.

## ABBREVIATIONS

3′UTR: 3′ untranslated region
5′UTR: 5′ untranslated region
Chr: chromosome
DMEM: dulbecco’s modified eagle medium
EGFR: epidermal growth factor receptor
H5N1: human avian influenza A
H1N1: human pandemic influenza A
HBV: hepatitis B virus
HCV: hepatitis C virus
HIV: human immunodeficiency virus
HPV: human papillomaviruses
HR: high-risk
LR: low-risk
miRNA: microRNAs
MUSCLE: multiple sequence comparison by log-expectation
ORF: open reading frame
PBS: phosphate buffered saline
PFV-1: primate foamy virus type 1
PI: propidium iodide
RTCA: Real time cell analysis
URR: upper regulatory region
